# Supplementary figures and images for: Motor Control of Distinct Layer 6 Corticothalamic Feedback Circuits
Source: eNeuro. 2024 Jul 4;11(7):ENEURO.0255-24.2024. doi: 10.1523/ENEURO.0255-24.2024 (PMC11236587; doi:10.1523/ENEURO.0255-24.2024)

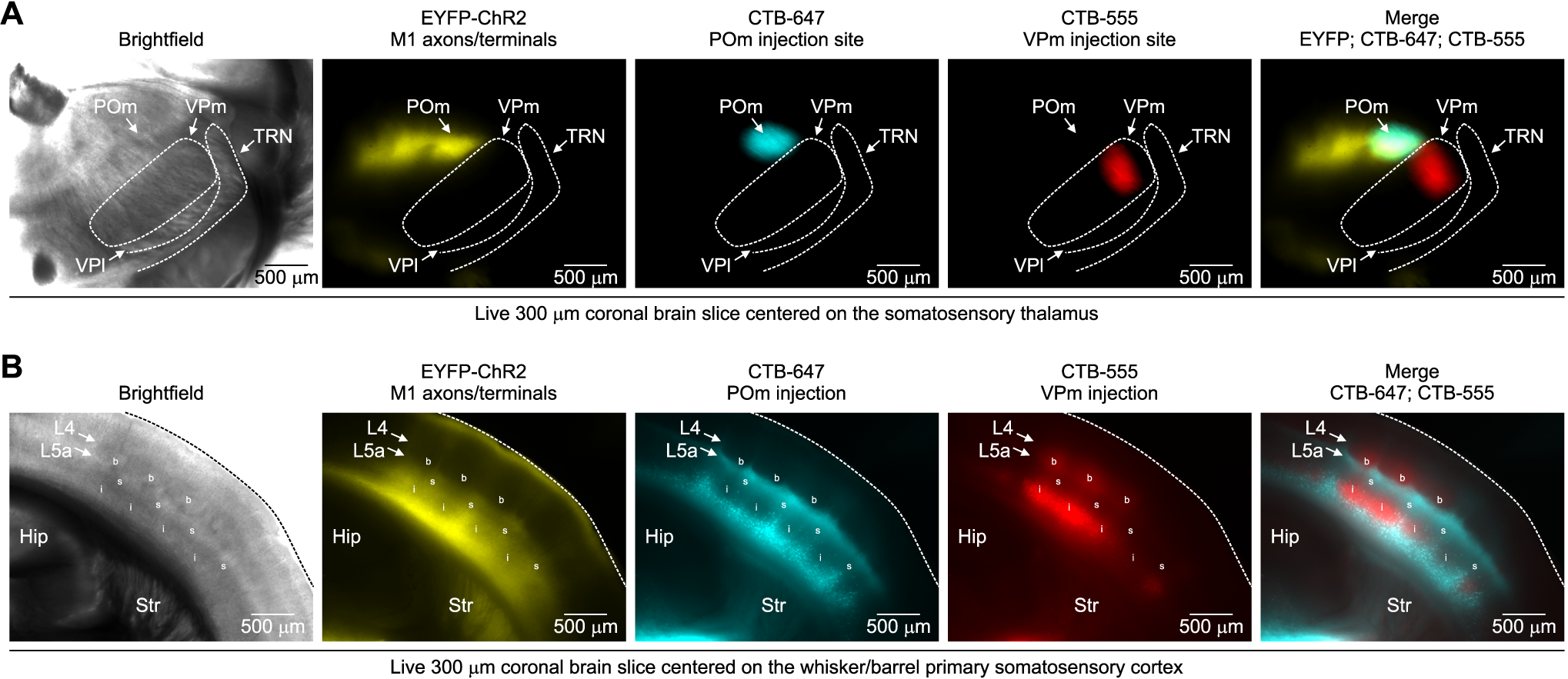

Supplement: Figure 3-1 — Confirming POm targeting based on M1 projections to the POm and the distribution of retrogradely labeled CT cells in L6a. A, Left, Brighfield image of a live coronal slice (300 μm) through somatosensory thalamus S1. Right, epifluorescence images of the same slice showing M1 axons/terminals in the thalamus (EYFP/ChR2) and the dual CTB injections (CTB-647 and CTB-555) into the POm and VPm. Note the overlap of EYFP and CTB-647 in the POm, confirming a successful hit. B, Left, Brighfield image of a live coronal slice (300 μm) through vibrissal/barrel primary somatosensory cortex (S1). Right, epifluorescence images of the same slice showing M1 axons/terminals in the S1 and distribution of retrogradely labeled cells from the POm (CTB-647) and VPm (CTB-555). POm, posterior medial nucleus; VPm, ventral posterior medial nucleus; VPl, ventral posterior lateral nucleus; TRN, thalamic reticular nucleus; Hip, hippocampus; Str, striatum; b, L4 barrel, s, septa; I, L6a infrabarrel. Download Figure 3-1, TIF file. [file eneuro-11-ENEURO.0255-24.2024-s001.tif]
